# *Fasciola gigantica* Recombinant Abelson Tyrosine Protein Kinase (r*Fg*Abl) Regulates Various Functions of Buffalo Peripheral Blood Mononuclear Cells

**DOI:** 10.3390/ani15020179

**Published:** 2025-01-10

**Authors:** Min Zhao, Yu Zou, Wanting Chen, Dongqi Wu, Chengjun Xian, Haoqing Yang, Jiacheng Tan, Wenda Di, Wende Wu, Dongying Wang

**Affiliations:** Guangxi Colleges and Universities Key Laboratory of Prevention and Control for Animal Disease, College of Animal Science and Technology, Guangxi University, Nanning 530005, China; zhaominzzzmmm@163.com (M.Z.); zyzy200002@126.com (Y.Z.); m19531343905@163.com (W.C.); wdq13695564874@163.com (D.W.); 18867029277@163.com (C.X.); 13408430348@163.com (H.Y.); 13350750821@163.com (J.T.); diwenda@gxu.edu.cn (W.D.); wwd0194@163.com (W.W.)

**Keywords:** *Fasciola gigantica*, Abelson tyrosine protein kinase, host–parasite interactions, immune regulation

## Abstract

*Fasciola gigantica* can modulate host immune mechanisms through excretory–secretory products (ESP). As one of the components of ESP, it is unknown whether Abelson tyrosine protein kinase (Abl) is involved in parasite–host immune interaction. To investigate the immunoregulatory function of Abl in *Fasciola gigantica*, we cloned and expressed the *Fasciola* gigantica Abl protein and assessed its effect on specific immune functions of buffalo peripheral blood mononuclear cells (PBMCs). Recombinant *F. gigantica* Abelson tyrosine protein kinase (r*Fg*Abl) was expressed in *Escherichia coli*. Western blot analysis was performed to assess the reactivity of anti-r*Fg*Abl antibodies with r*Fg*Abl, serum from *F. gigantica*-infected buffalo, and excretion and secretion products of *F. gigantica*. Immunohistochemical analysis was conducted to determine the localization of *Fg*Abl in tissues from larval stages and adult worms of *F. gigantica*. Furthermore, immunofluorescence analysis was utilized to evaluate the binding ability of the r*Fg*Abl protein to buffalo peripheral blood mononuclear cells (PBMCs), as well as to investigate the effects of varying concentrations of r*Fg*Abl protein (5, 10, 20, 40, and 80 μg/mL) on the functional responses of PBMCs. Anti-r*Fg*Abl antibodies specifically recognize r*Fg*Abl, serum from buffalo infected with *F. gigantica*, and *Fg*ESP. r*Fg*Abl is localized in the cecum and capsule of juvenile worms, as well as in the testis and viellaria of adult worms. Additionally, r*Fg*Abl enhances cell proliferation, migration, nitric oxide (NO) production, and phagocytosis, while also increasing the transcription levels of cytokines (IFN-γ, IL-12, TNF-α, IL-4, IL-10, and TGF-β). The results indicate that r*Fg*Abl can influence the immune function of PBMCs. Further investigation into the immunomodulatory properties of the r*Fg*Abl protein will enhance our understanding of the immune interaction mechanisms between trematodes and their hosts.

## Simple Summary

Abelson tyrosine protein kinase (Abl) has been demonstrated to influence the development of various helminths. However, as one of the components of the excretory secretory products (ESP), it remains unclear whether it affects the immunomodulatory mechanism of *F. gigantica*. To address this, we investigated the effects of *Fasciola gigantica* Abl protein on several immune functions of buffalo peripheral blood mononuclear cells (PBMCs). Our results indicate that Abl is a component of excretion and secretion products. The recombinant Abl protein enhances the proliferation, migration, nitric oxide (NO) production, and phagocytosis of PBMCs, while also upregulating the transcript levels of IFN-γ, IL-12, TNF-α, IL-4, IL-10, and TGF-β. These findings suggest that recombinant *F. gigantica* Abelson tyrosine protein kinase (r*Fg*Abl) plays a role in modulating the immune functions of PBMCs.

## 1. Introduction

Fascioliasis is a zoonotic parasitic disease primarily caused by *Fasciola hepatica* and *Fasciola gigantica*. The disease can infect a variety of mammals, including domestic animals, wild animals, and humans, with a notable prevalence in cattle, sheep, and goats [1,2]. Fascioliasis significantly impacts global public health and results in substantial socioeconomic losses, estimated at approximately USD 3.2 billion annually, and has been reported in 81 countries [3,4]. Triclabendazole (TCBZ) is a conventional method for the prevention and treatment of fascioliasis. However, due to its long-term and frequent use, more than 17 endemic countries have reported the development of resistance to TCBZ in *Fasciola* spp. [5,6]. To address the rising infection rates of *Fasciola* spp., the development of targeted vaccines represents a promising and sustainable approach. Comprehensive research into the interaction mechanisms between *Fasciola* spp. and its hosts will facilitate the identification of potential new targets for prevention and control.

During the invasion, migration, and maturation of *Fasciola*, the body integument and intestinal tract release excretory–secretory products (ESP) that contribute to immune evasion [7,8]. Various components of ESP have been shown to regulate the host immune response and facilitate long-term parasitism within the host organism, including peroxiredoxin, cathepsin L cysteine proteases and glutathione S-transferase [9,10,11]. In mammalian cells, protein kinases (PKs) are conserved signaling molecules that play crucial roles in various biological processes. PKs have been identified as high-potential drug targets for a range of parasitic helminths [12]. Among these, tyrosine protein kinase (PTK) has become an effective drug target for the treatment of human diseases and the development of anthelmintic strategies due to its key role in cell signaling pathways [13]. Abelson tyrosine protein kinase (Abl), a member of the PTK family, is widely expressed in various tissues and regulates numerous physiological activities, including cell proliferation and apoptosis, cytoskeletal remodeling, cell adhesion, stress response, and survival processes [14]. Currently, the role of Abl kinase in parasites is being increasingly studied. Its inhibitor, imatinib, has been shown to reduce motor activity and reproductive defects in *Schistosoma japonicum* [15]. Furthermore, Abl kinase inhibition has been confirmed to impact the intestinal, capsule, and gonadal development of *F. hepatica* [16]. Furthermore, it exerts adverse effects on other parasites, including *Schistosoma mansoni* [17] and *Toxoplasma gondii* [18]. While Abl has been demonstrated to negatively impact the development and reproduction of various parasites, its role in the pathogenesis of parasitic infections remains unclear.

The early transcriptomic results of *F. gigantica* indicated that Abl exhibited high transcript levels during the invasion stages of miracidia and metacercariae [19] and as one of the ESPs of *F. gigantica*, Abl may play a crucial role in the ability of *F. gigantica* to resist host immune responses. The aim of this study was to investigate whether *Fg*Abl is involved in the *F. gigantica*–host immune interaction and to extend the study of the effects of *Fg*ESPs components on host immune cell function. By cloning and expressing *Fg*Abl, we examined the effects of the recombinant protein *Fg*Abl (r*Fg*Abl) on various immune functions of buffalo PBMCs in vitro, including proliferation, migration, nitric oxide (NO) production, phagocytosis, phagocytosis, and cytokine secretion. Our data suggest that r*Fg*Abl affects multiple immune functions in buffalo PBMCs that are key components of the immunopathogenesis of *F. gigantica* infection.

## 2. Materials and Methods

### 2.1. Animals

Two 3-month-old female New Zealand rabbits and ten 6-week-old Kunming mice were acquired from the Experimental Animal Center of Guangxi Medical University and were raised in a temperature-controlled room at 25 ± 0.5 °C, fed ad libitum and provided with sufficient drinking water.

### 2.2. Source of Parasite

The adult *F. gigantica* was sourced from a slaughterhouse in Nanning City, Guangxi Province, where live *F. gigantica* eggs were collected and cultured in a 28 incubator, kept in the dark. The water was changed every three days. After 12 to 15 days of culture, miracidias were obtained within one hour of exposure to light. These miracidias were then used to infect Galba pervia (the intermediate host of *F. gigantica*), and metacercariaes were collected 35 to 37 days post-infection. Each Kunming mouse was orally administered 1 mL of PBS solution containing 15 metacercariaes through the sterile Gavage needle, and maintained at room temperature (25  ±  0.5 °C) with adequate food and free access to water. At 42 days post *F. gigantica* infection (dpi), the morphology and tissue structure of the *F. gigantica* juveniles were clearer in the mice. The mice were euthanized by intraperitoneal injection of sodium pentobarbital (0.1 mL/10 g), and the *F. gigantica* juveniles were collected. The serum from buffalo infected with *F. gigantica* was provided by the Parasitology Laboratory of the School of Animal Science and Technology at Guangxi University.

### 2.3. Cell Isolation and Culture

Peripheral venous blood samples were collected from three healthy buffaloes at the Buffalo Research Institute of the Guangxi Zhuang Autonomous Region, Chinese Academy of Agricultural Sciences. PBMCs were isolated and cultured according to established protocols [20]. Briefly, PBMCs were isolated using the PBMC isolation kit (TBD, Tianjin, China). The PBMCs were cultured in Roswell Park Memorial Institute 1640 (RPMI 1640) medium supplemented with 10% fetal bovine serum (FBS) and 1% penicillin-streptomycin (Gibco, New York, NY, USA), and maintained in a 5% CO_2_ atmosphere at 37 °C.

### 2.4. Cloning and Expression of Fasciola gigantica (Fg) Abelson Tyrosine Protein Kinase (Abl)

Total RNA was extracted from adult trematodes using Trizol, followed by reverse transcription into complementary DNA (cDNA) utilizing a cDNA synthesis kit (Vazyme, Nanjing, China). The cDNA was subsequently stored at −80 °C. Primers were designed using SnapGene, referencing the Abl gene sequence of *F. gigantica* in GenBank (GenBank: TPP58065.1). The *Fg*Abl gene was amplified using forward primer (5′-CGCGGATCCGGCGGAAACAGTGGAAAGTT-3′) and reverse primer (5′-CCGCTCGAGAACCAAATCAAACTCACCAAGCA-3′). The primers were designed to incorporate BamHI and XhoI restriction sites. The amplified *Fg*Abl gene product was ligated into pMD18-T using the pMD™18-T Vector Cloning Kit (Takara, Dalian, China). The resulting pMD18-T-*Fg*Abl plasmid was digested with BamH I and Xho I enzymes and subsequently cloned into pET28a. The pET28a-*Fg*Abl plasmid was transformed into *Escherichia coli* DH5α cells (Takara, Dalian, China). Identification was performed using double enzyme digestion and verification by sequencing (BGI Genomics, Guangzhou, China). The sequencing results were confirmed using the BLAST program (https://blast.ncbi.nlm.nih.gov/Blast.cgi (accessed on 17 January 2024)). The successfully cloned pET28a-*Fg*Abl plasmid was then introduced into *Escherichia coli* BL21 (DE3) cells.

### 2.5. Expression of Recombinant Fasciola gigantica (Fg) Abelson Tyrosine Protein Kinase (Abl) Protein

*Escherichia coli* BL21 (DE3) cells harboring the pET28a-*Fg*Abl recombinant plasmid were inoculated into LB medium to achieve an optimal optical density of 0.6 (OD600) at 37 °C. 1 mM Isopropyl-b-D-thiogalactopyranoside (IPTG) (Solarbio, Beijing, China) was added and the culture was incubated for an additional 10 h. Cells were harvested by centrifugation (12,000 rpm, 10 min, 4 °C), and the resulting cell pellet was sonicated at 250 W for 30 min (sonication for 30 s, pause for 15 s). The resulting mixture was then subjected to centrifugation at 10,000 rpm for 30 min. The sonicated mixture was centrifuged at 12,000 rpm for 20 min at 4 °C. Protein size was assessed using 12% (*w*/*v*) sodium dodecyl sulfate-polyacrylamide gel electrophoresis (SDS-PAGE).

### 2.6. Purification and Identification of Recombinant Fasciola gigantica (Fg) Abelson Tyrosine Protein Kinase (Abl) Protein

The sonicated precipitate was denatured in an 8 M urea buffer and purified using nickel column affinity chromatography at 4 °C. The purified r*Fg*Abl was dialyzed and renatured in buffers containing 8 M, 4 M, 2 M, 1 M, and 0.5 M urea, as well as PBS (pH 7.4). Protein concentration was determined using Bradford Protein Assay Kit (Solarbio, Beijing, China), with bovine serum albumin (BSA) serving as the standard. The purified r*Fg*Abl (20 μg) was separated by 12% SDS-PAGE and transferred to polyvinylidene fluoride (PVDF) membrane. The membranes were blocked with 5% skim milk buffer (TBST) for 2 h at 37 °C. The membranes were incubated with His-Tag (diluted 1:1000 in TBST) (Beyotime, Nanning, China) for 12 h at 4 °C. After incubation, we washed the membranes 5 times with TBST (5 min per wash) and used freshly prepared 3,3′-diaminobenzidine chromogenic substrate (Solarbio, Beijing, China).

### 2.7. Preparation of Polyclonal Antibodies and Western Blotting

Purified r*Fg*Abl (0.5 mg) was mixed with Freund’s complete adjuvant (1:1) and injected subcutaneously into New Zealand rabbits to induce the production of anti-r*Fg*Abl polyclonal antibodies. Every two weeks, equal proportions of Freund’s incomplete adjuvant and r*Fg*Abl were administered via the same route for booster immunization. After four immunizations, the rabbits were euthanized by intravenous injection of sodium pentobarbital (1 mL/kg) into the marginal ear. Blood samples were collected from rabbits and serum containing r*Fg*Abl antibody was isolated, which were stored at −80 °C until needed. The r*Fg*Abl, *Fg* native protein, and *Fg*ESP (20 μg) were separated by 12% SDS-PAGE, and transferred to PVDF membranes. Rabbit anti-r*Fg*Abl serum and normal rabbit serum were used as the primary antibodies (1:200 in TBST), and incubated for 12 h at 4 °C. Goat anti-rabbit IgG-HRP (Novus, Shanghai, China) (1:5000 dilution) was incubated at 37 °C for 2 h. Immunoreactions were detected utilizing 3,3′-diaminobenzidine as chromogenic substrate.

### 2.8. Tissue Localization of Abelson Tyrosine Protein Kinase (Abl) Protein in F. gigantica

Juveniles (42 dpi) of *F. gigantica* and adults were collected and fixed in paraformaldehyde for 24 h. The worms were dehydrated using a series of alcohol gradients, and the tissues were embedded in paraffin. Paraffin blocks were sectioned for antigen retrieval, followed by soaking in 0.01 M citrate buffer (pH 6.0) and heating at 95 °C for 10 min. After cooling, the sections were washed three times with PBS solution, with each wash lasting 5 min. The blocks were then treated with 5% bovine serum albumin and incubated for 15 min. The samples were incubated overnight at 4 °C with rabbit anti-*Fg*Abl and normal rabbit serum at a dilution of 1:200. The sections were then incubated with Cy3-labeled goat anti-rabbit IgG (Beyotime, Nanning, China) at 37 °C for 1 h. Finally, DAPI (Beyotime, Nanning, China) was added, and the samples were incubated at room temperature for 15 min before being observed under laser confocal microscope (Nikon A1R, Nikon Instruments Inc., Tokyo, Japan).

### 2.9. Immunofluorescence Detection of Recombinant Fasciola gigantica (Fg) Abelson Tyrosine Protein Kinase (Abl) Protein Binding to Buffaloe Peripheral Blood Mononuclear Cells (PBMCs)

Immunofluorescence assay (IFA) was performed according to previous studies [21]. Briefly, we added 1 mL of PBMC, r*Fg*Abl protein and PBS for the blank control group to the 12-well plate. We incubated the cells for 6 h at 37 °C in a 5% CO_2_ incubator. We washed the cells five times with PBS and fixed the cells with paraformaldehyde for 30 min at 37 °C. We washed the cells five times with PBS and blocked with 5% BSA in PBS at 37 °C for 1 h. We incubated the rabbit anti-r*Fg*Abl polyclonal antibody with the r*Fg*Abl-treated and PBS group PBMCs for 12 h at 4 °C. Subsequently, we incubated them with Cy3-labeled goat anti-rabbit IgG (1:500 dilution) for 1 h at 37 °C. We stained the samples with DAPI and analyzed the samples using Zeiss laser scanning microscope (LSM710, Zeiss, Jena, Germany).

### 2.10. The Effect of Recombinant Fasciola gigantica (Fg) Abelson Tyrosine Protein Kinase (Abl) Protein on Cell Viability

A total of 100 μL of PBMCs at density of 10^6^ cells were added to 96-well cell plate. Introduced PBS as control, along with concanavalin A (10 μg/mL), and a series of r*Fg*Abl concentrations (5, 10, 20, 40, and 80 μg/mL) into the corresponding wells. The plate was incubated in the 37 °C, 5% CO_2_ incubator for 48 h. In total, 10 μL of CCK-8 reagent (Beyotime, Nanning, China) was added to each well and allowed to incubate in the dark for the additional 4 h. Measured the absorbance at 450 nm (OD450) using a microplate reader (Bio-Rad Laboratories, Hercules, CA, USA). We calculated the cells proliferation index using the following formula: OD450 (r*Fg*Abl)/OD450 (PBS group).

### 2.11. Determination of Nitric Oxide

In total, 1 mL of PBMCs at a density of 10^6^ cells, along with PBS and r*Fg*Abl (5, 10, 20, 40, and 80 μg/mL), were added to 12-well cell plate. The cells were cultured in a 5% CO_2_ atmosphere at 37 °C for 24 h. The cells supernatant was collected by centrifugation at 4500 rpm for 5 min. The Total Nitric Oxide Assay Kit (Beyotime, Nanning, China) was employed to detect nitric oxide (NO) levels. Absorbance at 540 nm (OD540) was measured using a microplate reader (Bio-Rad Laboratories, Hercules, CA, USA), and NO levels were calculated based on a standard curve.

### 2.12. The Effect of Recombinant Fasciola gigantica (Fg) Abelson Tyrosine Protein Kinase (Abl) Protein on Cell Migration

PBMCs were seeded into 24-well cell plates at 10^6^ cells per well, and various concentrations of r*Fg*Abl were added to stimulate the cells. The plates were incubated at 37 °C with 5% CO_2_ for 24 h. The cells were collected and the concentration was adjusted to 10^5^ cells/mL. The Transwell chamber (Beyotime, Nanning, China) was placed into the cell plate, and 100 μL of PBMCs (10^5^ cells/mL) was added to the top chamber. After 4 h of incubation, the cells in the lower chamber were counted using a Neubauer counting chamber.

### 2.13. Effect of Recombinant Fasciola gigantica (Fg) Abelson Tyrosine Protein Kinase (Abl) Protein on Phagocytic Activity

Flow cytometry was employed to assess the uptake of FITC-dextran by PBMCs, thereby evaluating their phagocytic activity. PBMCs at 10^6^ cells per well were stimulated with varying concentrations of r*Fg*Abl (5, 10, 20, 40, and 80 μg/mL) for 24 h in a 37 °C incubator with 5% CO_2_. Following stimulation, the cell supernatant was discarded, and the cells were washed three times with sterile PBS. In total, 100 μL of FITC-dextran (Thermo Fisher, Waltham, MA, USA) was added, and the cells were incubated in the dark for 1 h. After incubation, the cells were collected via centrifugation (2000 rpm, 5 min), and their phagocytic capacity was evaluated using FACSAria Flow Cytometer (BD Biosciences, San Jose, CA, USA), with the results analyzed using FlowJo 10.

### 2.14. Cytokine Analysis

PBMCs at a concentration of 10^6^ cells per well were incubated with varying concentrations of r*Fg*Abl in 37 °C, 5% CO_2_ incubator for 48 h. Following incubation, cells were collected, and cellular RNA was extracted using TRIZOL reagent and reverse transcribed into cDNA. LightCycler^®^ 96 Instrument (Roche, Basel, Switzerland) was utilized to detect the expression levels of interleukin-4 (IL-4), IL-10, IL-12, interferon gamma (IFN-γ), transforming growth factor beta (TGF-β), and tumor necrosis factor alpha (TNF-α), along with reference gene Glyceraldehyde-3-phosphate dehydrogenase (GAPDH) (Supplementary Materials: Table S1) The cycling conditions were as follows: initial denaturation at 95 °C for 30 s; amplification at 95 °C for 10 s and 60 °C for 30 s and a melting curve stage at 60–95 °C. Three biological replicates were conducted for each sample.

### 2.15. Statistical Analysis

Sample data were analyzed using either the Dunnett test or the t-test, employing the GraphPad Prism 9.5 software package (GraphPad Software, San Diego, CA, USA). A difference was deemed statistically significant when *p* < 0.05. Data are presented as mean ± standard deviation (SD) [22].

## 3. Results

### 3.1. RFgAbl Expression, Purification, and Identification

**Figure 1:**
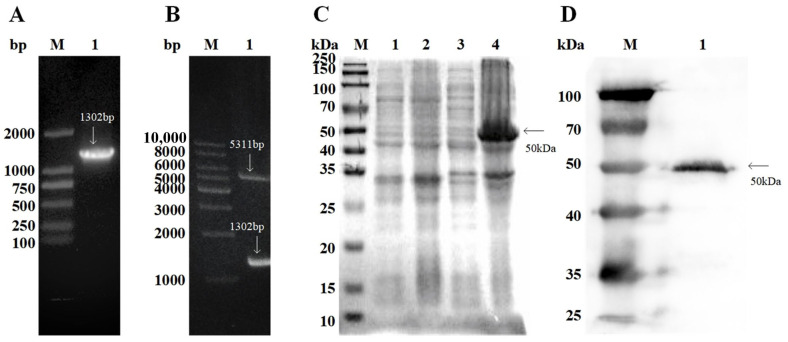
Cloning, expression, and Western blot analysis of *Fasciola gigantica* (*Fg*) Abelson tyrosine protein kinase (Abl). (**A**,**B**) M: DNA molecular weight standard. (**A**) 1: Amplification of the Abl gene from *F. gigantica* cDNA. (**B**) 1: Double enzyme restriction digest of the rpET28a-*Fg*Abl plasmid. (**C**,**D**) M: Protein molecular weight standard. (**C**) 1: Supernatant of bacterial cell lysate prior to induction; 2: Pellet of bacterial cell lysate prior to induction; 3: Supernatant of cell lysate induced at 37 °C for 10 h; 4: Cell lysates were pelleted after induction at 37 °C for 10 h. (**D**) 1: r*Fg*Abl was transferred to PVDF membrane and recognized by the His tag antibody.

As shown in Figure 1, the *Fg*Abl gene was amplified using RT-PCR, resulting in the detection of a 1302 bp band via gel electrophoresis. The recombinant plasmid pET28a-*Fg*Abl was confirmed through double enzyme digestion with BamHI and XhoI, which yielded 1302 bp and 5311 bp bands observed on gel electrophoresis. The gene fragment was successfully cloned into the pET-28a vector. Isopropyl-β-d-thiogalactopyranoside (IPTG) was employed to induce protein expression in the *Escherichia coli* BL21 (DE3) strain. The r*Fg*Abl is a His-tagged fusion protein, primarily produced as inclusion bodies. Following purification, a protein with a molecular weight of 50 kDa was detected using SDS-PAGE. Western blot analysis confirmed that the purified r*Fg*Abl was recognized by the His tag antibody.

### 3.2. Polyclonal Antibody Specificity

**Figure 2:**
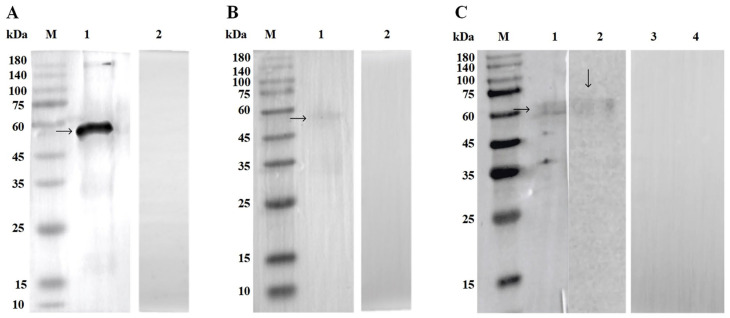
Western blot analysis of r*Fg*Abl. (**A**–**C**) M: protein molecular weight standard; (**A**) 1: *Fg*Abl recombinant protein, which was detected by rabbit anti-r*Fg*Abl serum, revealing a band at approximately 50 kDa; 2: *Fg*Abl recombinant protein. No bands were observed in serum incubation of healthy rabbits (**B**) 1: *Fg*Abl recombinant protein reacted with serum from fluke-infected buffalo, resulting in the detection of a band at ~50 kDa; 2: *Fg*Abl recombinant protein. *Fg*Abl recombinant protein. Healthy bovine serum incubation is band-free. (**C**) 1: *F. gigantica* native protein reacted with rabbit anti-r*Fg*Abl serum, yielding a band at ~50 kDa; 2: *Fg*ESP reacted with rabbit anti-r*Fg*Abl serum, also detecting a band at ~50 kDa. 3-4: *Fg* natural protein and *Fg*ESP. Incubation with healthy rabbit serum without bands. The final form of the image is stitched together.

Western blot analysis was employed to assess the specificity of rabbit anti-r*Fg*Abl polyclonal antibodies. As shown in Figure 2, anti-r*Fg*Abl antibodies produced in New Zealand white rabbits were capable of reacting with r*Fg*Abl, *Fg* native protein, and *Fg*ESP. In contrast, no reactivity was detected in the sera of unimmunized New Zealand rabbits. The specificity of the r*Fg*Abl protein was further confirmed through Western blot analysis, demonstrating that the r*Fg*Abl protein could react with the serum of buffaloes infected with *F. gigantica*.

### 3.3. Abl Immunofluorescence Localization in F. gigantica

**Figure 3:**
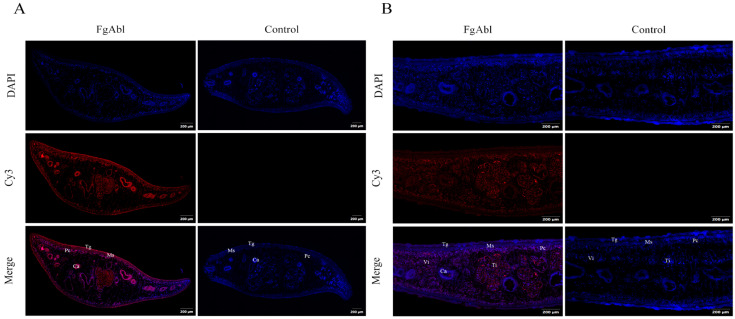
Distribution of *Fg*Abl in juvenile (42 dpi) and adult worms. Tg: tegument; Ms: muscle; Pc: parenchyma; Ca: cecum; Ti: Testis. Vi: viellaria. (**A**) The localization of *Fg*Abl protein in juvenile worms (42 dpi) is observed in the capsule, muscles, and cecal epithelium. (**B**) The *Fg*Abl protein in adult worms is primarily located in the testis and viellaria. Red indicates the target antigen stained by a Cy3-coupled secondary antibody, while blue represents the nucleus stained by DAPI. The term ‘Merge’ refers to the combined effect of DAPI and Cy3 staining. Scale bars represent 200 μm.

Twenty-two juveniles (42 dpi) were collected from mice infected with metacercariae, and juveniles with intact worms were selected for tissue immunofluorescence experiments. The distribution of *Fg*Abl protein in juveniles (42 dpi) of *F. gigantica* and in adult tissues was detected using immunofluorescence. As shown in Figure 3, in juvenile worms, *Fg*Abl protein is predominantly localized in the capsule and cecal epithelium; in adult worms, *Fg*Abl is primarily found in the testes.

### 3.4. Binding Affinity of rFgAbl Protein to Buffaloe Peripheral Blood Mononuclear Cells (PBMCs)

**Figure 4:**
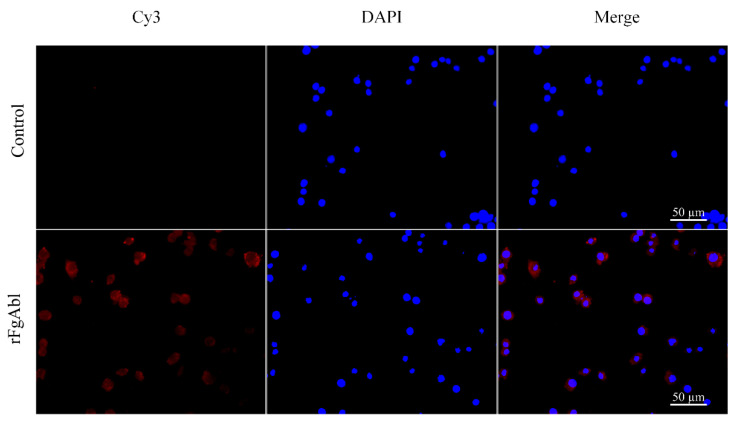
The recombinant *F. gigantica*Abelson tyrosine protein kinase (r*Fg*Abl) binds to the surface of buffalo peripheral blood mononuclear cells (PBMCs). PBMCs treated and untreated with r*Fg*Abl were incubated with rabbit anti-r*Fg*Abl antibody and stained with Cy3-conjugated goat anti-rabbit IgG. PBMCs surface staining was observed in cells treated with r*Fg*Abl, whereas no staining was detected in untreated cells. Scale bar: 50 μm.

The binding affinity of buffalo PBMCs to the r*Fg*Abl protein was evaluated using an indirect immunofluorescence assay. Following incubation with rabbit anti-r*Fg*Abl and Cy3-labeled goat anti-rabbit IgG (which emits red fluorescence), as shown in Figure 4, red complexes were detected on the surface of the PBMCs, while DAPI-stained nuclei exhibited blue fluorescence. No red fluorescence was observed in the untreated control group.

### 3.5. RFgAbl Promotes the Proliferation and Migration of Buffalo Peripheral Blood Mononuclear Cells (PBMCs)

**Figure 5:**
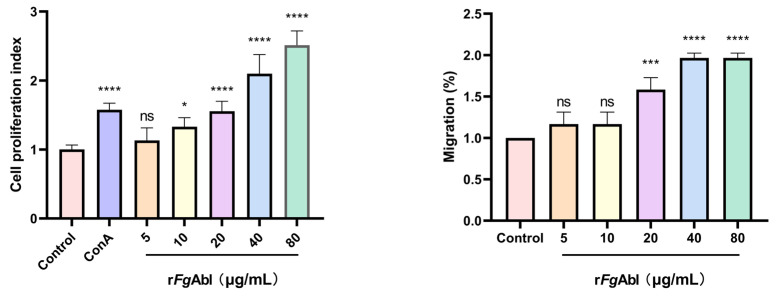
r*Fg*Abl promotes the proliferation and migration of buffalo PBMCs. Buffalo PBMCs were treated with phosphate-buffered saline (PBS), concanavalin A (10 μg/mL), and various concentrations of r*Fg*Abl (μg/mL) before being incubated at 37 °C for 48 h. Cell proliferation was assessed using the CCK-8 method. The results indicated that r*Fg*Abl significantly enhanced the proliferation of PBMCs. PBMCs were treated with phosphate-buffered saline (PBS) and various concentrations of r*Fg*Abl (μg/mL) to assess the percentage of cell migration (%). Graphs represent means ± standard deviations of data from 3 independent biological replicates; Asterisks denote significant differences between control and treated cells (* *p* < 0.05; *** *p* < 0.001; **** *p* < 0.0001; ns, not significant).

As shown in Figure 5, treatment with concentrations of 10 μg/mL, 20 μg/mL, 40 μg/mL, and 80 μg/mL significantly enhanced cell proliferation compared to the control group, but the 5 μg/mL did not. (ConA: ANOVA, *F*_(6, 35_) = 60.34, *p* < 0.0001; 5 μg/mL: ANOVA, *F*_(6, 35_) = 60.34, *p* = 0.8363; 10 μg/mL: ANOVA, *F*_(6, 35)_ = 60.34, *p* = 0.0287; 20 μg/mL: ANOVA, *F*_(6, 35)_ = 60.34, *p* < 0.0001; 40 μg/mL: ANOVA, *F*_(6, 35)_ = 60.34, *p* < 0.0001; 80 μg/mL: ANOVA, *F*_(6, 35)_ = 60.34, *p* < 0.0001.) Similarly, concentrations of 20 μg/mL, 40 μg/mL, and 80 μg/mL improved cell migration in comparison to the control group, while the 5 μg/mL and 10 μg/mL concentrations did not (5 μg/mL: ANOVA, *F*_(5, 12)_ = 47.41, *p* = 0.4457; 10 μg/mL: ANOVA, *F*_(5, 12)_ = 47.41, *p* = 0.4457; 20 μg/mL: ANOVA, *F*_(5, 12)_ = 47.41, *p* = 0.0003; 40 μg/mL: ANOVA, *F*_(5, 12)_ = 47.416*, p* < 0.0001; 80 μg/mL: ANOVA, *F*_(5, 12)_ = 47.41, *p* < 0.0001).

### 3.6. RFgAbl Promotes NO Production and Cellular Phagocytosis of Buffaloe Peripheral Blood Mononuclear Cells (PBMCs)

**Figure 6:**
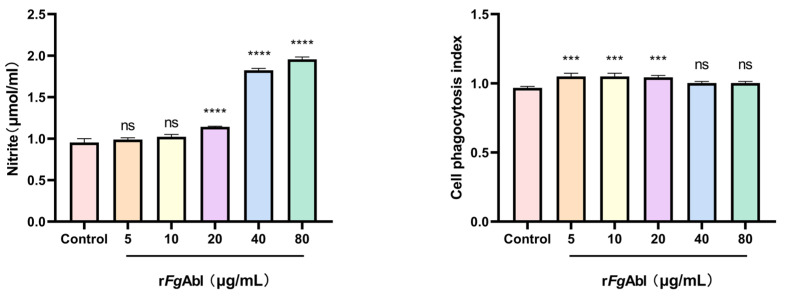
Effect of r*Fg*Abl on NO Production and Cellular phagocytosis in PBMCs. After treating PBMCs with PBS and various concentrations of r*Fg*Abl (μg/mL) for 24 h, the total concentration of NO produced by the PBMCs was measured using the Griess method. PBMCs were treated with PBS and varying concentrations of r*Fg*Abl (μg/mL), after which the cellular uptake of FITC-dextran was measured. Graphs represent means ± standard deviations of data from 3 independent biological replicates; Asterisks denote significant differences between control and treated cells (*** *p* < 0.001; **** *p* < 0.0001; ns, not significant).

As shown in Figure 6, the release of NO from r*Fg*Abl-treated PBMCs was significantly increased at concentrations of 20 μg/mL, 40 μg/mL and 80 μg/mL, while no increase was observed at concentrations of 5 μg/mL and 10 μg/mL (5 μg/mL: ANOVA, *F*_(5, 12)_ = 774.3, *p* = 0.6321; 10 μg/mL: ANOVA, *F*_(5, 12)_ = 774.3, *p* = 0.0943; 20 μg/mL: ANOVA, *F*_(5, 12)_ = 774.3, *p* < 0.0001; 40 μg/mL: ANOVA, *F*_(5, 12)_ = 774.3, *p* < 0.0001; 80 μg/mL: ANOVA, *F*_(5, 12)_ = 774.3, *p* < 0.0001). Phagocytosis was assessed through the uptake of fluorescein isothiocyanate (FITC)-dextran. Following stimulation of PBMCs with r*Fg*Abl, flow cytometry was employed to evaluate the phagocytic activity of the cells, r*Fg*Abl significantly enhanced the phagocytosis of PBMCs at concentrations of 10 μg/mL, 20 μg/mL, and 40 μg/mL; however, no significant changes were observed at concentrations of 5 μg/mL and 80 μg/mL (5 μg/mL: ANOVA, *F*_(5, 12_) = 12.52, *p* = 0.0003; 10 μg/mL: ANOVA, *F*_(5, 12)_ = 12.52, *p* = 0.0003; 20 μg/mL: ANOVA, *F*_(5, 12)_ = 12.52, *p* = 0.0005; 40 μg/mL: ANOVA, *F*_(5, 12)_ = 12.52, *p* = 0.0964; 80 μg/mL: ANOVA, *F*_(5, 12)_ = 12.52, *p* = 0.0964).

### 3.7. RFgAbl Modulation of Buffaloe Peripheral Blood Mononuclear Cells (PBMCs) Cytokines

**Figure 7:**
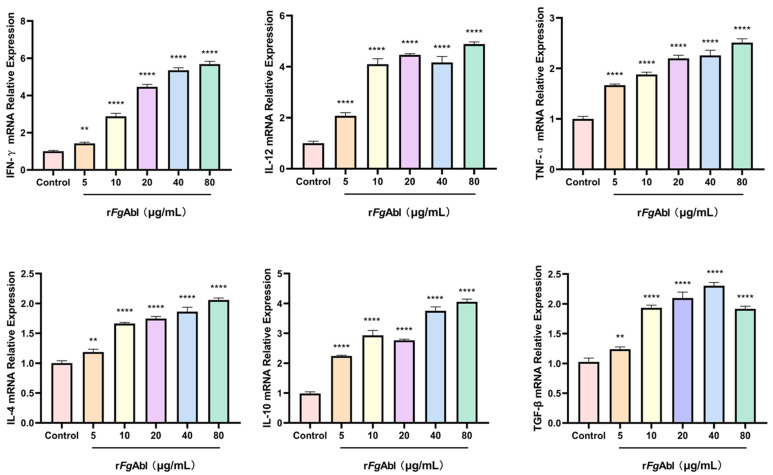
Effect of r*Fg*Abl on cytokines in PBMCs. After incubating PBMCs with r*Fg*Abl for 48 h, the transcription levels of IFN-γ, IL-12, TNF-α, IL-4, IL-10, and TGF-β were measured using real-time fluorescence PCR. Graphs represent means ± standard deviations of data from 3 independent biological replicates. Asterisks indicate significant differences between control and treated cells (** *p* < 0.01; **** *p* < 0.0001).

As shown in Figure 7, in comparison to the control group, all concentrations of *Fg*Abl (5 μg/mL, 10 μg/mL, 20 μg/mL, 40 μg/mL, and 80 μg/mL) resulted in increased levels of IFN-γ, IL-12, TNF-α, IL-4, IL-10, and TGF-β (IFN-γ (5 μg/mL: ANOVA, *F*_(5, 12)_ = 780.4, *p* = 0.0052; 10 μg/mL: ANOVA, *F*_(5, 12)_ = 780.4, *p* < 0.0001; 20 μg/mL: ANOVA, *F*_(5, 12)_ = 780.4, *p* < 0.0001; 40 μg/mL: ANOVA, *F*_(5, 12)_ = 780.4, *p* < 0.0001; 80 μg/mL: ANOVA, *F*_(5, 12)_ = 780.4, *p* < 0.0001), IL-12 (5 μg/mL: ANOVA, *F*_(5, 12)_ = 327.2, *p* < 0.0001; 10 μg/mL: ANOVA, *F*_(5, 12)_ = 327.2, *p* < 0.0001; 20 μg/mL: ANOVA, *F*_(5, 12)_ = 327.2, *p* < 0.0001; 40 μg/mL: ANOVA, *F*_(5, 12)_ = 327.2, *p* < 0.0001; 80 μg/mL: ANOVA, *F*_(5, 12)_ = 327.2, *p* < 0.0001), TNF-α (5 μg/mL: ANOVA, *F*_(5, 12)_ = 208.8, *p* < 0.0001; 10 μg/mL: ANOVA, *F*_(5, 12)_ = 208.8, *p* < 0.0001; 20 μg/mL: ANOVA, *F*_(5, 12)_ = 208.8, *p* < 0.0001; 40 μg/mL: ANOVA, *F*_(5, 12)_ = 208.8, *p* < 0.0001; 80 μg/mL: ANOVA, *F*_(5, 12)_ = 208.8, *p* < 0.0001), IL-4 (5 μg/mL: ANOVA, *F*_(5, 12)_ = 251.6, *p* = 0.0011; 10 μg/mL: ANOVA, *F*_(5, 12)_ = 251.6, *p* < 0.0001; 20 μg/mL: ANOVA, *F*_(5, 12)_ = 251.6, *p* < 0.0001; 40 μg/mL: ANOVA, *F*_(5, 12)_ = 251.6, *p* < 0.0001; 80 μg/mL: ANOVA, *F*_(5, 12)_ = 251.6, *p* < 0.0001); IL-10 (5 μg/mL: ANOVA, *F*_(5, 12)_ = 385.2, *p* < 0.0001; 10 μg/mL: ANOVA, *F*_(5, 12)_ = 385.2, *p* < 0.0001; 20 μg/mL: ANOVA, *F*_(5, 12)_ = 385.2, *p* < 0.0001; 40 μg/mL: ANOVA, *F*_(5, 12)_ = 385.2, *p* < 0.0001; 80 μg/mL: ANOVA, *F*_(5, 12)_ = 385.2, *p* < 0.0001); TGF-β (5 μg/mL: ANOVA, *F*_(5, 12)_ = 196.3, *p* = 0.0049; 10 μg/mL: ANOVA, *F*_(5, 12)_ = 196.3, *p* < 0.0001; 20 μg/mL: ANOVA, *F*_(5, 12)_ = 196.3, *p* < 0.0001; 40 μg/mL: ANOVA, *F*_(5, 12)_ = 196.3, *p* < 0.0001; 80 μg/mL: ANOVA, *F*_(5, 12)_ = 196.3, *p* < 0.0001).

## 4. Discussion

During the evolutionary process, *Fasciola* spp. has developed a series of complex immune evasion mechanisms to evade attacks by the host immune system. Transcriptomic analyses of *F. gigantica* revealed that *Fg*Abl exhibited high transcript levels in the metacercarial stage, indicating that *Fg*Abl may play a critical role in regulating host immunity. This study amplified the Abl gene of *Fg*Abl using RT-PCR and successfully constructed the *Fg*Abl prokaryotic expression vector. Western blotting confirmed that the rabbit anti-r*Fg*Abl antibody can interact with both r*Fg*Abl and native *F. gigantica* proteins. Additionally, r*Fg*Abl was recognized by the serum of buffaloes infected with *F. gigantica*. The transcript levels of Abl varied across different developmental stages of *F. gigantica*, suggesting that Abl may serve different biological functions at various stages of its life cycle. Immunofluorescence results indicated that the *Fg*Abl protein was present in the capsule and cecal epithelium of juveniles (42 dpi) of *F. gigantica*, confirming that *Fg*Abl is an excretory and secretory protein. In adult worms, *Fg*Abl was primarily localized in the testis and viellaria, which may be associated with the reproductive processes of the worms. Furthermore, r*Fg*Abl specifically binds to buffalo PBMCs, implying that *Fg*Abl may play a role in regulating the immune function of host PBMCs.

When pathogens invade, Th1 cells secrete IFN-γ to promote cellular immunity, while Th2 cells enhance humoral immunity by producing IL-4, IL-5, and IL-10. In the early stages of *Fasciola* spp. infection, the immune response is typically characterized as a mixed Th1/Th2 type. However, as the duration of the infection increases, this response gradually shifts towards a Th2 dominance, leading to the suppression of Th1 activity. This shift is advantageous for the long-term survival of the parasite within the host and mitigates host tissue damage [23,24]. Similar to r*Fg*Rab10 [20] and r*Fg*CatB [25], when r*Fg*Abl was cultured with PBMC, transcript levels of Th1-type cytokines (IFN-γ, IL-12, TNF-α) and Th2-type cytokines (IL-4, IL-10 and TGF-β) were increased. The cytokines IFN-γ and TNF-α can promote the release of NO and upregulate oxygen free radicals, enhancing phagocytosis [26,27,28]. NO secretion and phagocytosis increased after r*Fg*Abl treatment, which is similar to previous studies on the r*Fg* Rab10 protein [20] and may be due to increased transcript levels of IFN-γ and TNF-α. However, unlike the r*Fg*Rab10 protein, r*Fg*Abl can also promote cell proliferation and migration. The increase in cell proliferation increases the number of immune cells involved, and the increase in migration allows immune cells to reach the parasite site in time. This shows that r*Fg*Abl activates cells and enhances the host’s immune defense. In the serum of goats infected with *F. hepatica*, the level of TNF-α gradually increased throughout the infection process. In addition to inducing worm cell death, TNF-α appears to play a role in the liver injury associated with *F. hepatica* infection. Furthermore, IL-12 induces IFN-γ secretion in worms and downregulates Th2 cell populations, thereby enhancing Th1-type responses [29]. For long-term survival within the host, *Fasciola* spp. and its immune regulatory molecules downregulate the inflammatory response in a number of ways, including inducing Th2 cells to secrete the anti-inflammatory cytokine IL-4, which contributes to the repair and resolution of helminth-induced tissue damage. IL-4 can also promote B cell differentiation and the production of anti-parasite specific antibodies Ig G1 and Ig E, thereby exerting humoral immunity to control parasitic infections [30]. IL-4 promotes the secretion of IL-10 and TGF-β, which are considered key mediators in mediating immunosuppression [31]. In another study (r*Fg*14-3-3e), IL-10 and TGF-β secretion can cause immunosuppression [32]. Upregulation of IL-10 may have a negative impact on IFN-γ production, thereby suppressing Th1 immune responses and creating a favorable environment for parasite survival [33]. We observed significant increases in IFN-γ, IL-12, and TNF-α, indicating that r*Fg*Abl may induce Th1-type responses. IL-4, IL-10 and TGF-β were also significantly increased, especially IL-4 (a key cytokine in Th2 immunity), indicating that r*Fg*Abl may also induce Th2 immunity at the same time, that is, showing Th1/Th2 mixed immunity.

## 5. Conclusions

Our research findings indicate that r*Fg*Abl interacts with the serum of buffaloes infected with *F. gigantica*, and immunofluorescence studies reveal its localization in the cecum of juvenile worms, confirming that *Fg*Abl is an excretory secretion product. Additionally, r*Fg*Abl is capable of binding to buffalo PBMCs. Following treatment with r*Fg*Abl, we observed enhanced proliferation and migration of PBMCs, along with increased NO secretion and phagocytic activity. And increased the transcription levels of cytokines IFN-γ, IL-12, TNF-α, IL-4, IL-10 and TGF-β, indicating that *Fg*Abl plays regulatory role in the immune interaction between the *F. gigantica* and host. Although r*Fg*Abl has some regulatory effect on PBMC, the key molecules and immunoregulatory mechanisms that interact with *Fg*Abl are still unknown and require further research.

## Data Availability

The data presented in this study are available on request from the corresponding author.

## Supplementary Materials

The following supporting information can be downloaded at: https://www.mdpi.com/article/10.3390/ani15020179/s1, Figure S1: Cloning, Expression, and Western blot Analysis of *Fg*Abl. Figure S2: Western blot analysis of r*Fg*Abl. Figure S3: Distribution of *Fg*Abl in 42(dpi) juvenile and adult worms. Figure S4: r*Fg*Abl binds to the surface of buffalo PBMCs. Figure S5: Proliferation and Migration. Figure S6: Production and Cellular phagocytosis. Figure S7: Effect of r*Fg*Abl on cytokines in PBMCs. Table S1: Sets of Primer for GADPH and *Fg*Abl used in transcription analysis by real-Time PCR.
