# Correction: Zhao et al. *Fasciola gigantica* Recombinant Abelson Tyrosine Protein Kinase (r*Fg*Abl) Regulates Various Functions of Buffalo Peripheral Blood Mononuclear Cells. *Animals* 2025, *15*, 179

**DOI:** 10.3390/ani15040553

**Published:** 2025-02-14

**Authors:** Min Zhao, Yu Zou, Wanting Chen, Dongqi Wu, Chengjun Xian, Haoqing Yang, Jiacheng Tan, Wenda Di, Wende Wu, Dongying Wang

**Affiliations:** Guangxi Colleges and Universities Key Laboratory of Prevention and Control for Animal Disease, College of Animal Science and Technology, Guangxi University, Nanning 530005, China; zhaominzzzmmm@163.com (M.Z.); zyzy200002@126.com (Y.Z.); m19531343905@163.com (W.C.); wdq13695564874@163.com (D.W.); 18867029277@163.com (C.X.); 13408430348@163.com (H.Y.); 13350750821@163.com (J.T.); diwenda@gxu.edu.cn (W.D.); wwd0194@163.com (W.W.)

**Error in Figure 4**

In the original publication [1], there was a mistake in Figure 4 as published. Some of the unpublished materials (fluorescent image of FgBMP-1) were included in the figure which should have been removed. There was a mistake in the Figure 4 legend as published. Some of the unpublished materials (fluorescent image of FgBMP-1) were included in the figure which should have been removed. The corrected Figure 4 appears below.

**Author Name and Order Change**

In the published publication, there was an error regarding the name of Yu Zou. The order of the authors has been updated as follows: Min Zhao, Yu Zou, Wanting Chen, Dongqi Wu, Chengjun Xian, Haoqing Yang, Jiacheng Tan, Wenda Di, Wende Wu and Dongying Wang. 

**Text Correction**

There was an error in the original publication. Some of the unpublished materials were included in the text which should have been removed.

1. A correction has been made to the Materials and Methods Section, *2.9. Immunofluorescence Detection of Recombinant Fasciola gigantica (Fg) Abelson Tyrosine Protein Kinase (Abl) Protein Binding to Buffaloe Peripheral Blood Mononuclear Cells (PBMCs)*, first paragraph:

“Immunofluorescence assay (IFA) was performed according to previous studies [21]. Briefly, we added 1 mL of PBMC, r*Fg*Abl protein and PBS for the blank control group to the 12-well plate. We incubated the cells for 6 h at 37 °C in a 5% CO_2_ incubator. We washed the cells five times with PBS and fixed the cells with paraformaldehyde for 30 min at 37 °C. We washed the cells five times with PBS and blocked with 5% BSA in PBS at 37 °C for 1 h. We incubated the rabbit anti-r*Fg*Abl polyclonal antibody with the r*Fg*Abl-treated and PBS group PBMCs for 12 h at 4 °C.”

2. Results Section, *3.4. Binding Affinity of rFgAbl Protein to Buffaloe Peripheral Blood Mononuclear Cells (PBMCs)*, first paragraph:

“Following incubation with rabbit anti-r*Fg*Abl and Cy3-labeled goat anti-rabbit IgG (which emits red fluorescence), as shown in Figure 4, red complexes were detected on the surface of the PBMCs, while DAPI-stained nuclei exhibited blue fluorescence. No red fluorescence was observed in the untreated control group.”

**References**

With this correction, one of the references has been removed (reference 21—unpublished materials) and replaced with another one: Ehsan, M.; Hu, R.S.; Hou, J.L.; Elsheikha, H.M.; Li, X.D.; Liang, P.H.; Zhu, X.Q. *Fasciola gigantica* tegumental calcium-binding EF-hand protein 4 exerts immunomodulatory effects on goat monocytes. *Parasit. Vectors* **2021**, *14*, 276.

The authors state that the scientific conclusions are unaffected. This correction was approved by the Academic Editor. The original publication has also been updated.

## Figures and Tables

**Figure 4:**
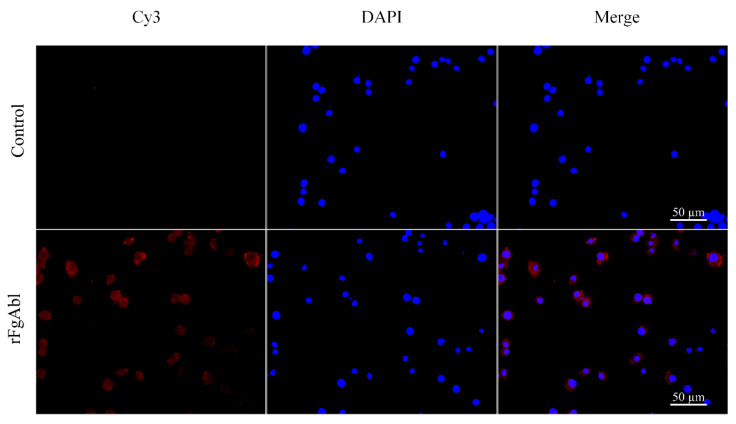
The recombinant *F. gigantica*Abelson tyrosine protein kinase (r*Fg*Abl) binds to the surface of buffalo peripheral blood mononuclear cells (PBMCs). PBMCs treated and untreated with r*Fg*Abl were incubated with rabbit anti-r*Fg*Abl antibody and stained with Cy3-conjugated goat anti-rabbit IgG. PBMCs surface staining was observed in cells treated with r*Fg*Abl, whereas no staining was detected in untreated cells. Scale bar: 50 μm.
