# Supplementary material for: Fasciola gigantica Recombinant Abelson Tyrosine Protein Kinase (rFgAbl) Regulates Various Functions of Buffalo Peripheral Blood Mononuclear Cells
Source: Animals (Basel). 2025 Jan 10;15(2):179. doi: 10.3390/ani15020179 (PMC11758316; doi:10.3390/ani15020179)
Supplement: Supplementary file 1 [file animals-15-00179-s001.zip › Animal Experimental Ethical Inspection Form of Guangxi University.pdf]

附件

广西大学动物实验伦理审查表

Animal Experimental Ethical Inspection Form of Guangxi University

编号（No）：GXU-2023-0099

<table>
<tr><td rowspan="9">申请人填写的相关信息 (Related information filled by applicant)</td><td colspan="2">申请单位
Name of organization</td><td colspan="2">广西大学</td><td colspan="2">申请人
Applicant</td><td>王冬英</td></tr>
<tr><td colspan="2">申请人学历
Education of applicant</td><td colspan="2">研究生</td><td colspan="2">技术职称
Professional title</td><td>副教授</td></tr>
<tr><td colspan="2">实验名称
Experiment title</td><td colspan="5">磷酸化、去磷酸化调控大片形吸虫发育和免疫逃避的机制研究</td></tr>
<tr><td rowspan="4">拟进动物情况</td><td>动物来源
Source of animal</td><td colspan="5">BALB/c 小鼠，实验家兔</td></tr>
<tr><td>品种品系
Species of strain</td><td>BALB/c 小鼠，实验家兔，健康 水牛 5 头</td><td>等级
Grade</td><td>清 洁 级</td><td>规格
Specifications</td><td>BALB/c 小鼠：6-8 周龄
兔：80 日龄</td></tr>
<tr><td>数量
Number</td><td>小鼠♂ 80 只、♀ 70 只，家兔♂ 4 只，水牛♂ 5 头</td><td>申请日期
Application date</td><td colspan="3">2023.03.06</td></tr>
<tr><td>进驻日期
Entering date</td><td>2023.06</td><td>结束日期
Ending date</td><td colspan="3">2027.12</td></tr>
<tr><td colspan="7">实验要点，包括实验目的、实验方法、观测指标、实验结束后处死动物的方法等(Aim of experiment , Outline of experiments, experimental methods, observational index, executing animal method, et al)</td></tr>
<tr><td colspan="7">为研究磷酸化、去磷酸化调控大片形吸虫发育和免疫逃避机制，拟从实验动物中心购买BALB/c 小鼠，实验家兔，每只小鼠实验感染 10 个囊蚴，感染后 6 周依据动物伦理相关程序处死，解剖，采集肝脏，收集虫体。应用重组表达大片形吸虫 PP2A 蛋白免疫家兔，采集血清制备多克隆抗体，实验结束后依据动物伦理相关程序处死。实验结束后的小鼠和兔的尸体集中冻存，由学校统一安排清运及处理。选择健康水牛，无菌采集颈静脉抗凝血分离外周血单个核细胞用于实验研究，采血后水牛由其主人继续养殖。</td></tr>
<tr><td></td><td colspan="2">申请人签名
Signature of applicant</td><td>王冬英</td><td>联系电话
Telephone</td><td>办公室(O)
移 动(M)</td><td colspan="2">07713235635
15907817914</td></tr>
</table>

（请翻看背面）

<table>
<tr><td>申请者声明
Announcement of applicant</td><td colspan="5">我将自觉遵守实验动物福利伦理原则，随时接受实验动物伦理委员会的监督与检查，如违反规定，自愿接受处罚。(I will abide by the rules of animal experimental ethics, accept the supervision and inspection of the animal experimental ethics committee, and accept the punishment if any infringement.)

声明人签名(Signature)：王会荣

2023 年 3 月 14 日</td></tr>
<tr><td>审查依据
Inspection contents</td><td colspan="5">1. 该项目是否必须用实验动物进行实验，即能否用计算机模拟、细胞培养等非生命方法替代动物或用低等动物替代高等动物进行实验(Does laboratory animal must be used in the project? Could other methods such as computer simulation. cell culture or using the low-grade animal instead of the high－grade animal?)
2. 表中所填申请人资格和所用动物的品种品系、质量等级、规格是否合适，能否通过改良设计方案或用高质量的动物来减少所用动物的数量(Are the qualification of applicant，species or strain，grade and specifications of animals suitable? Could the quantity of animals be reduced by improving the study design or using high quality animals?)
3. 能否通过改进实验方法、调整实验观测指标、改良处死动物的方法，来优化实验方案、善待动物(Could the study design and animal treatment be refined by ameliorating experimental method，adjusting observational index. executing animal method?)</td></tr>
<tr><td rowspan="2">审查结果
Results of inspection</td><td>课题负责人意见
Project director attitude</td><td>同意
Agree ☑</td><td>不同意
Disagree □</td><td>签名
Signature</td><td>王会荣</td></tr>
<tr><td>实验动物伦理委员会意见
Attitude of the Animal Care & Welfare Committee</td><td>同意
Agree ☑</td><td>不同意
Disagree □</td><td colspan="2" rowspan="2">马健

签章(Stamp)

2023 年 3 月 15 日</td></tr>
<tr><td colspan="4">备注(Remark)

[stamp: 西大学 动物实验伦理审查委员会]</td></tr>
</table>

说明：

1. 编号由实验动物伦理委员会秘书填写。
2. 表格所有填写内容请用签字笔填写或电脑打印(签名处除外)。项目负责人、执行人及合作单位负责人均需在声明人签字栏签字。
3. 需随本表递交相关审查资料如实验方案、课题标书等。要求写明项目的意义、必要性、项目中有关实验动物的用途、饲养管理或实验处置方法、预期出现的对动物的伤害、处死动物的方法、项目进行中涉及动物福利和伦理问题的详细描述。
4. 此表一式 3 份，申请人、伦理委员会及科技处各存 1 份。

本表归档人员：李金鑫（签名）；　本表归档时间：2023.3.15
