# Supplementary material for: Fasciola gigantica Recombinant Abelson Tyrosine Protein Kinase (rFgAbl) Regulates Various Functions of Buffalo Peripheral Blood Mononuclear Cells
Source: Animals (Basel). 2025 Jan 10;15(2):179. doi: 10.3390/ani15020179 (PMC11758316; doi:10.3390/ani15020179)

Fasciola gigantica recombinant Abelson tyrosine protein kinase (_Fg_Abl) regulates various functions of buffalo peripheral blood mononuclear cells

Supplementary Figure:

Fig 1. A: Amplification of the Abl gene from *F. gigantica* cDNA.

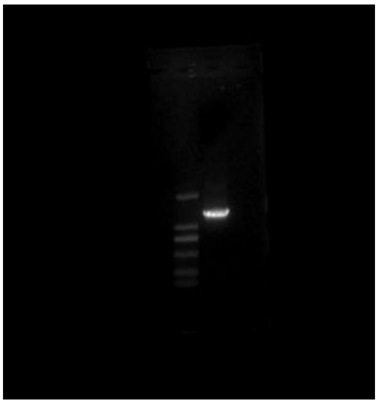

Fig 1. B: Double enzyme restriction digest of the rpET28a-*Fg*Abl plasmid.

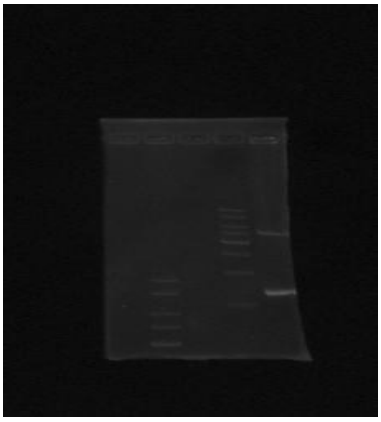

Fig 1. C: Supernatant of bacterial cell lysate prior to induction.

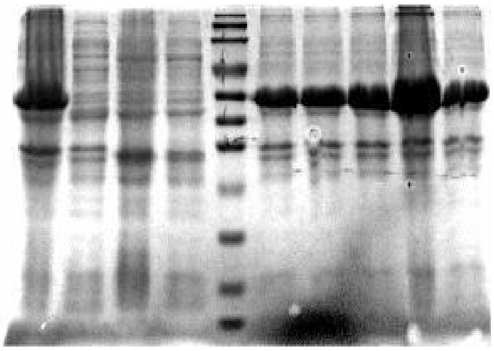

Fig 1. D: r*Fg*Abl was transferred to PVDF membrane and recognized by the His tag antibody.

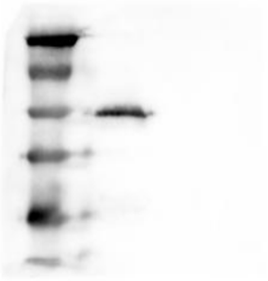

Fig 2. A: r*Fg*Abl with rabbit anti-r*Fg*Abl serum and rabbit normal serum.

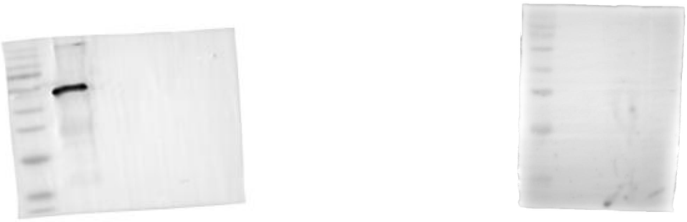

Fig 2. B: r*Fg*Abl with *F. gigantica*-infected buffalo serum and buffalo normal serum.

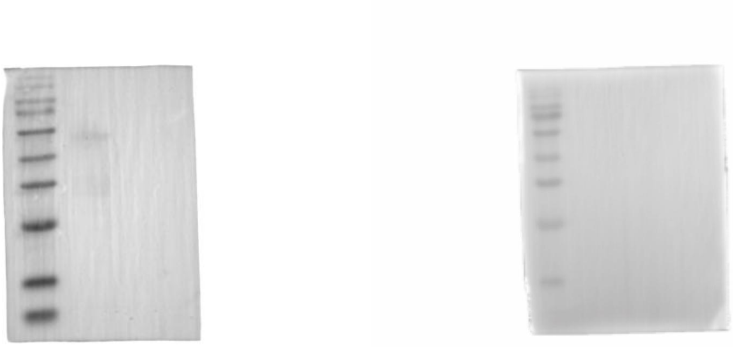

Fig 2. C: *Fg* natural protein, *Fg*ESP with rabbit anti-r*Fg*Abl serum and rabbit normal serum.

Fg natural protein *Fg*ESP rabbit normal serum

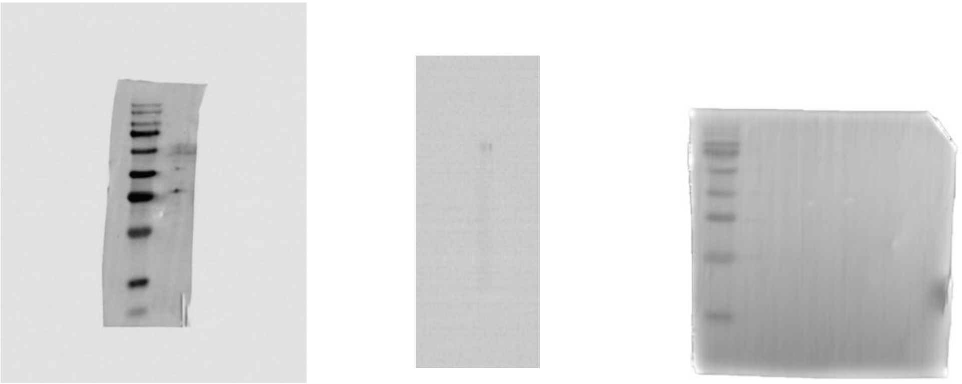

Fig 3. A: *Fg*Abl protein is localised at 42 dpi in child worm tissue.

r*Fg*Abl Merge

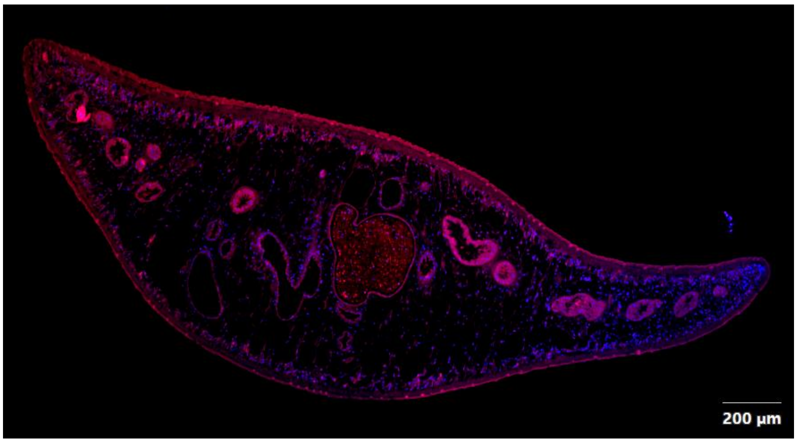

r*Fg*Abl DAPI

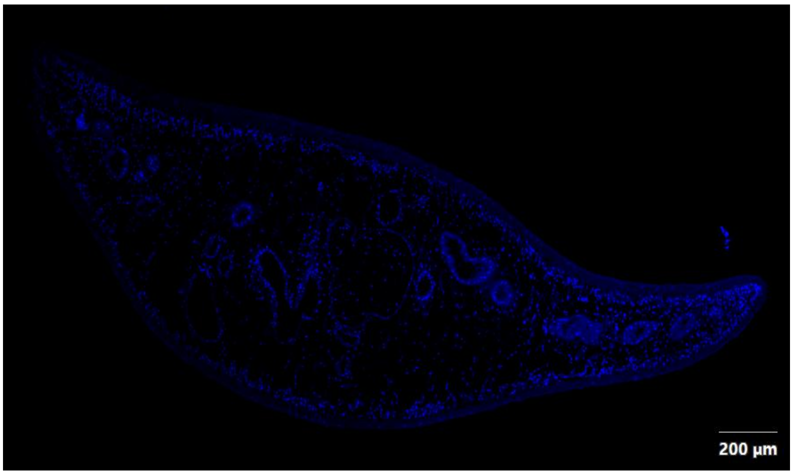

r*Fg*Abl Cy3

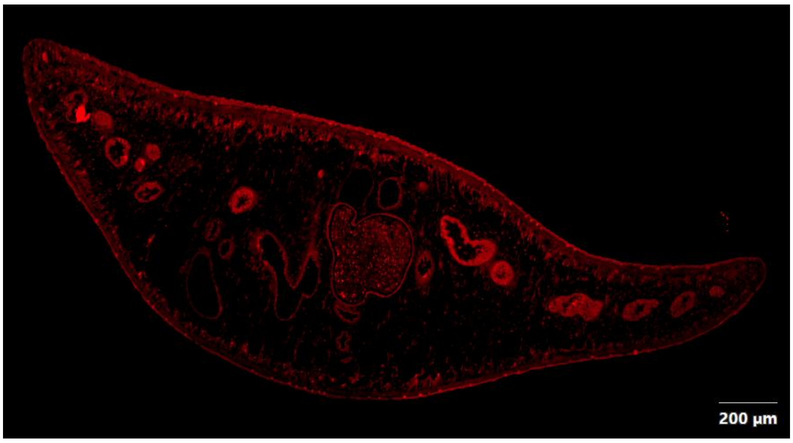

Control Merge

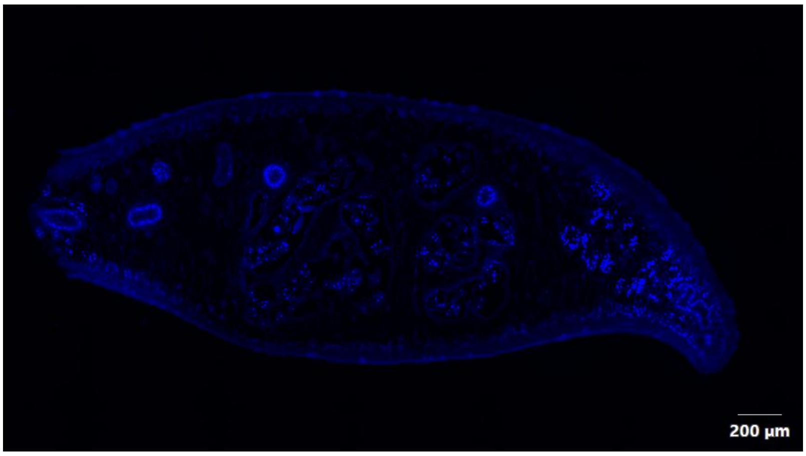

Control DAPI

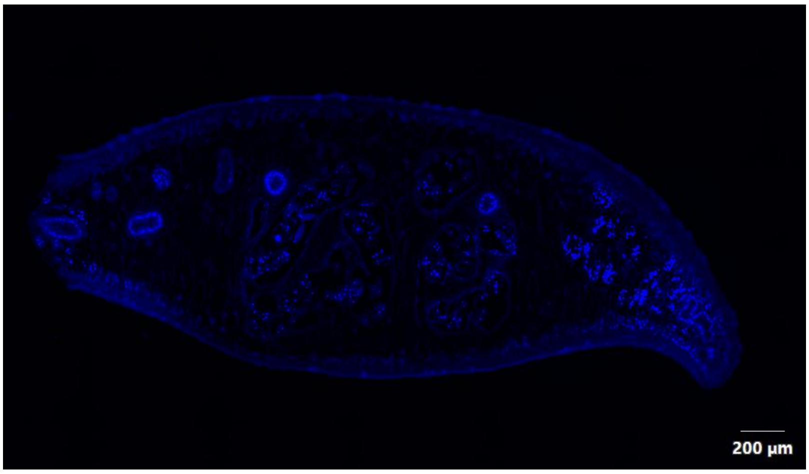

Control Cy3

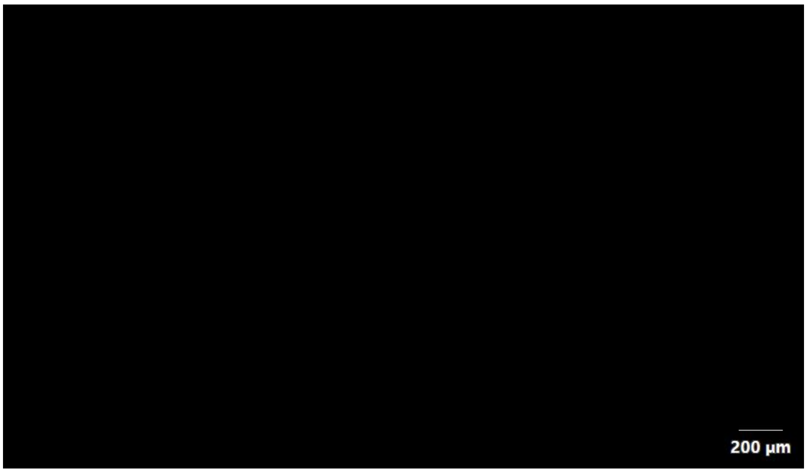

Fig 3. B: *Fg*Abl protein localisation in adult worm tissues.

r*Fg*Abl Merge

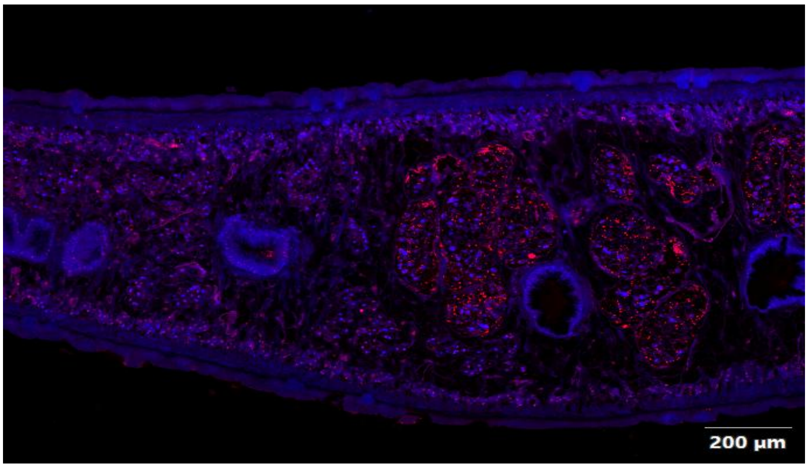

r*Fg*Abl DAPI

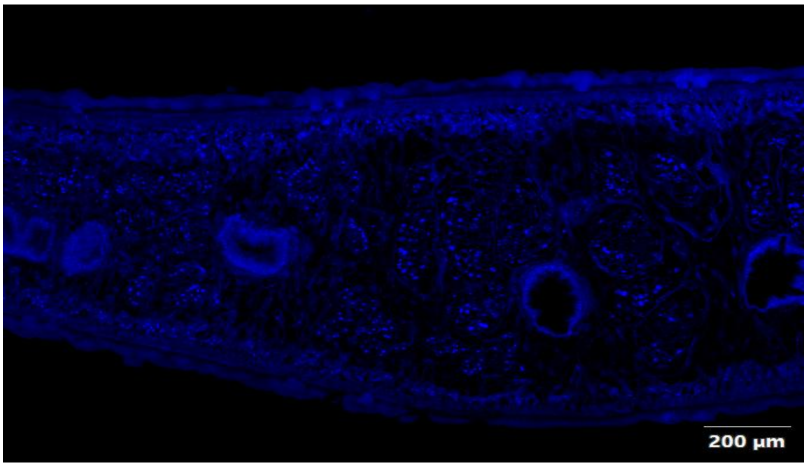

r*Fg*Abl Cy3

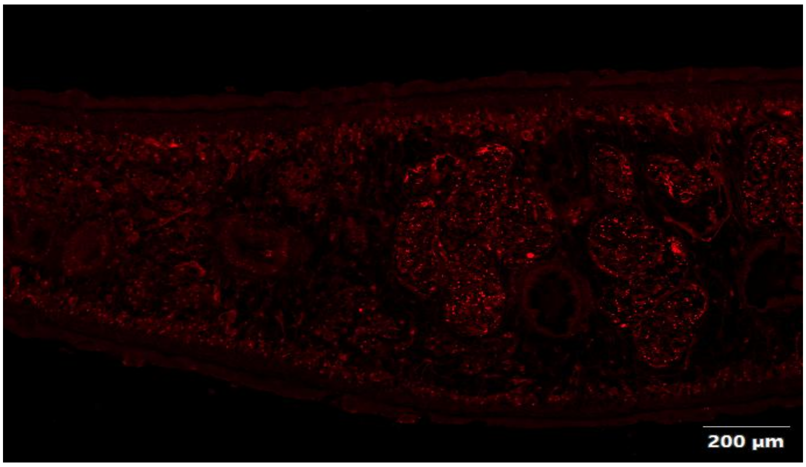

Control Merge

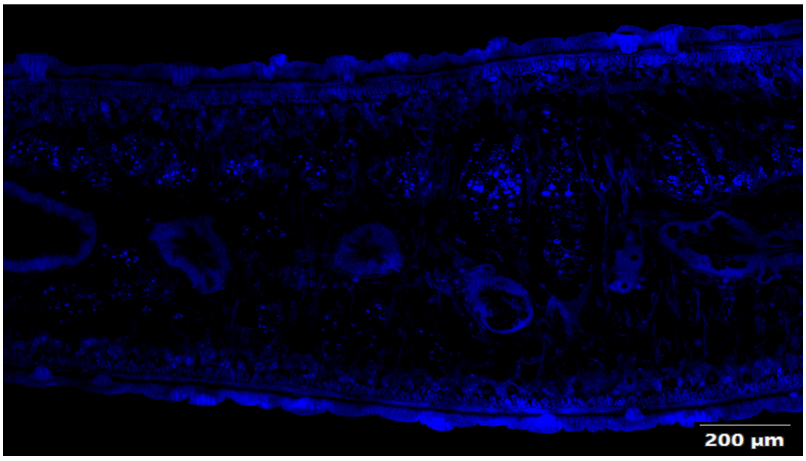

Control DAPI

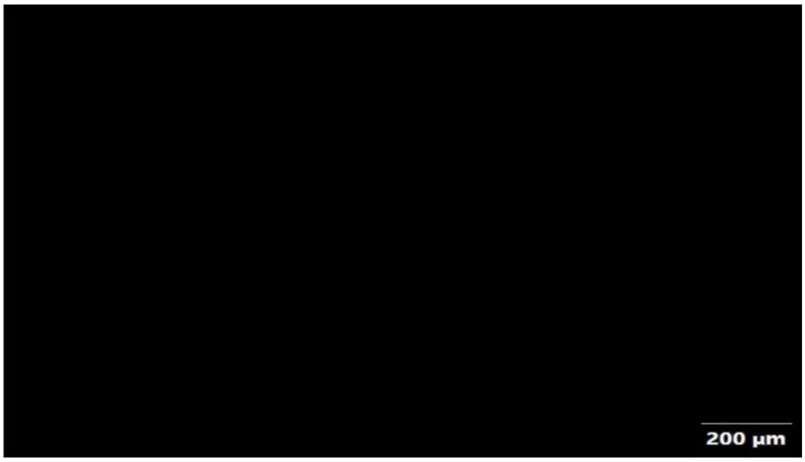

Control Cy3

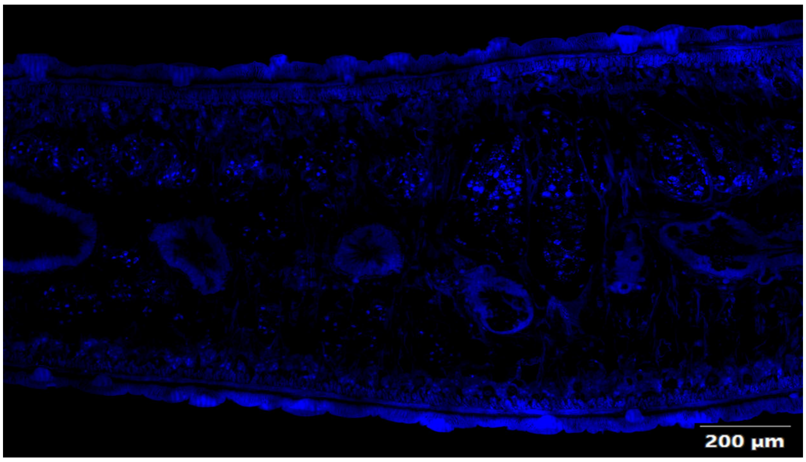

Fig 4. r*Fg*Abl binds to the surface of buffalo PBMCs

r*Fg*Abl

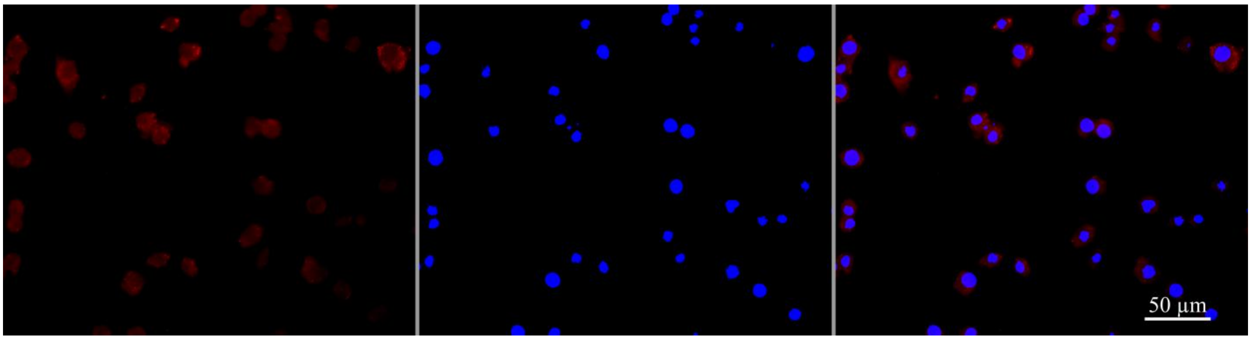

r*Fg*BMP-1

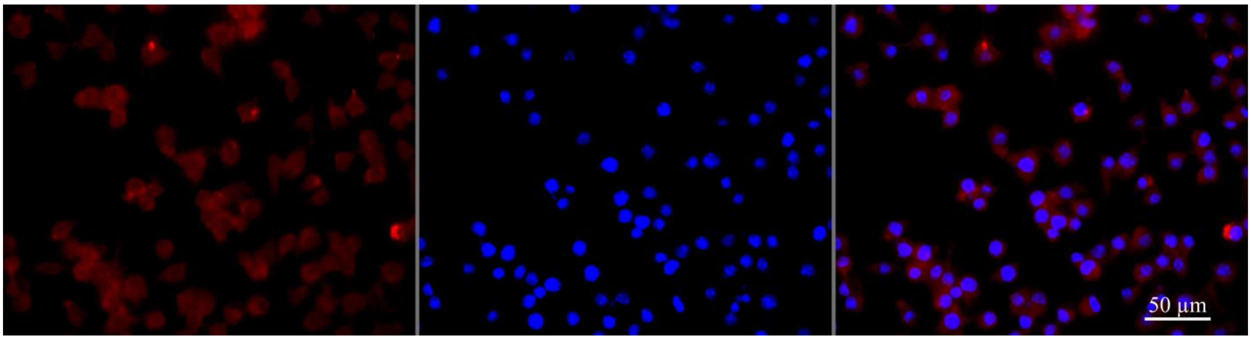

Control

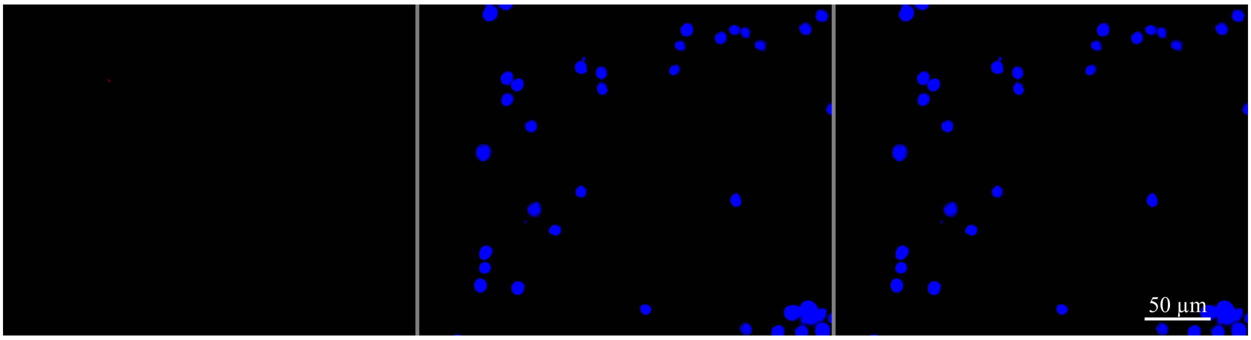

Supplement: Supplementary file 1 [file animals-15-00179-s001.zip › Original Images for Blots.pdf]
