# Supplementary material for: Fasciola gigantica Recombinant Abelson Tyrosine Protein Kinase (rFgAbl) Regulates Various Functions of Buffalo Peripheral Blood Mononuclear Cells
Source: Animals (Basel). 2025 Jan 10;15(2):179. doi: 10.3390/ani15020179 (PMC11758316; doi:10.3390/ani15020179)
Supplement: Supplementary file 1 [file animals-15-00179-s001.zip › Table 1. Sets of Primer for GADPH and FgAbl used in transcription analysis by .pdf]

Fasciola gigantica recombinant Abelson tyrosine protein kinase (_Fg_Abl) regulates various functions of buffalo peripheral blood mononuclear cells

Supplementary Table:

Table 1. Sets of Primer for GADPH and FgAbl used in transcription analysis by real-Time PCR.

Gene Name			Pre-amplified fragment size/bp
GADPH	GADPH-F	5'-CCTGCACCACCAACTGCTTG-3'	222
	GADPH-R	5'-TTGAGCTCAGGGATGACCTT-3'	
IL-4	IL-4-F	5'-CAGCATGGAGCTGCCT-3'	117
	IL-4-R	5'-ACAGAACAGGTCTTGCTTGC-3'	
IL-10	IL-10-F	5'-CTGTGCCTCTCCCCTAGAGT-3'	236
	IL-10-R	5'-GCAGCTAGCTCCACAAGGAA-3'	
IFN-ϒ	IFN-ϒ-F	5'-GTCTCCTTCTACTTCAAACT-3'	253
	IFN-ϒ-R	5'-ATTCTGACTTCTCTTCCGCT-3'	
TGF-β	TGF-β-F	5'-CGTGCTAATGGTGGAATAC-3'	208
	TGF-β-R	5'-GCCAGGAATTGTTGCTATA-3'	
IL-12	IL-12-F	5'-CAGGGACATCATCAAACCAG-3'	214
	IL-12-R	5'-CTTGTGGCATGTGACTTTGG-3'	
TNF-α	TNF-α-F	5'-ACACTGAACTGAGCCATC-3'	121
	TNF-α-R	5'-GCAAGGGCTCTTGATGGCAGA -3'	
